# Beyond the Heart: The Significance of Depression in Cardiac Surgery

**DOI:** 10.1093/ejcts/ezaf277

**Published:** 2025-08-08

**Authors:** Malin Stenman, Veronica Jackson, Josefin Särnholm, Anna Falk, Susanne J Nielsen, Ulrik Sartipy

**Affiliations:** Department of Molecular Medicine and Surgery, Karolinska Institutet, 17177, Stockholm, Sweden; Perioperative Medicine and Intensive Care Function, Karolinska University Hospital, 17176, Stockholm, Sweden; Department of Molecular Medicine and Surgery, Karolinska Institutet, 17177, Stockholm, Sweden; Stockholm Healthcare Services, Region Stockholm, 10431, Stockholm, Sweden; Department of Clinical Neuroscience, Division of Psychology, Karolinska Institutet, 17177, Stockholm, Sweden; Center for Behavioral Cardiovascular Health, Columbia University Irving Medical Center, New York, NY, 10027-6907, United States; Perioperative Medicine and Intensive Care Function, Karolinska University Hospital, 17176, Stockholm, Sweden; Department of Physiology and Pharmacology, Karolinska Institutet, 17177, Stockholm, Sweden; Department of Molecular and Clinical Medicine, Sahlgrenska Academy, Gothenburg University, 40530, Gothenburg, Sweden; Department of Cardiothoracic Surgery, Sahlgrenska University Hospital, 41345, Gothenburg, Sweden; Department of Molecular Medicine and Surgery, Karolinska Institutet, 17177, Stockholm, Sweden; Department of Cardiothoracic Surgery, Karolinska University Hospital, 17176, Stockholm, Sweden

**Keywords:** depression, cardiac surgery, cardiovascular disease, cardiac psychiatry, cognitive behavioral therapy

## Abstract

**Objectives:**

Preoperative depression is common among patients with cardiovascular disease and a significant risk factor for worse outcomes after cardiac surgery. This review summarizes the current evidence on depression as a risk factor and possible treatment options in patients undergoing cardiac surgery, and highlights future perspectives for clinical research.

**Methods:**

This narrative review was based on a selection of key papers, identified through collegial expert discussions rather than a systematic literature search.

**Results:**

Depression is often underdiagnosed and undertreated in patients undergoing cardiac surgery, significantly affecting recovery and increasing the risk of adverse outcomes. Implementing systematic psychological screening for depression and anxiety preoperatively and during follow-up is crucial for identifying at-risk patients. Psychological interventions, especially cognitive behavioral therapy have been shown to offer substantial benefits. Adopting a multidisciplinary approach integrating cardiovascular and psychological care is essential for improving recovery and long-term outcomes. Incorporating psychological screening and interventions into standard care can enhance postoperative outcomes, reduce complications, and provide comprehensive support for cardiac surgery patients.

**Conclusions:**

Depression is often overlooked in cardiac surgery patients, despite its significant impact on recovery and long-term outcomes. Systematic psychological screening before and after surgery together with a multidisciplinary approach integrating cardiovascular and psychological care can improve patient outcomes. Future research should clarify how depression and cardiovascular disease are linked, assess treatment effectiveness, and identify appropriate intervention strategies.

## Graphical abstract

**Figure:**
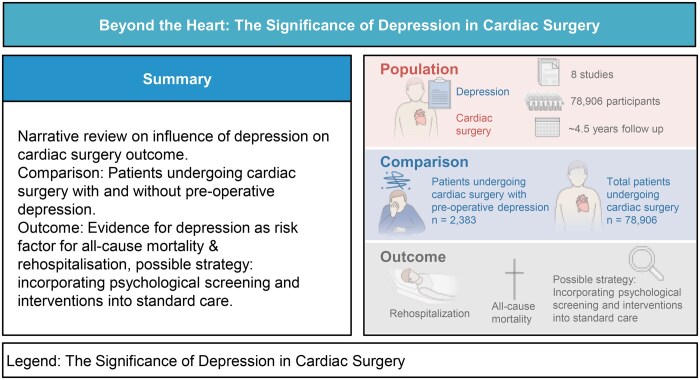

## INTRODUCTION

Cardiovascular disease (CVD), remains the leading cause of morbidity and mortality among men and women worldwide.^1^ Major depressive disorder (MDD) is a widespread condition, impacting about 10% of the population.^2^ Women are about twice as likely as men to experience depression at some point in their lives.^3^ MDD is a growing global issue and has been consistently linked to an increased risk of CHD. Depression is significantly more common among CHD patients, occurring 2-3 times more frequently than in the general population.^4^ The prevalence of depression among patients with CHD ranges from 15% to 30% and is, like in the general population, approximately twice as high in women compared to men.^3^ This established link between depression and CHD identifies depression as a risk factor for CHD. Preoperative depression has been observed in up to 60% of patients undergoing cardiac surgery and is associated with higher rates of mortality, readmissions, wound infections, and postoperative cardiac events.^5^^,^^6^

We conducted a narrative review to report the latest data on the association between depression and prognosis after cardiac surgery and to discuss proposed mechanisms (physiological, behavioural, and societal) underlying the association between depression and prognosis after cardiac surgery.

## METHODS

We conducted a targeted literature review to present the latest findings on the link between preoperative depression and outcomes after cardiac surgery. Our search included PubMed and Google Scholar to find relevant original research and review articles. We focused on terms related to epidemiology (eg, “cardiovascular disease depression prevalence”), the relationship between depression and postoperative outcomes (eg, “depression cardiac surgery mortality rehospitalizations postoperative delirium”), pathophysiological and health behaviour mechanisms (eg, “depression inflammation,” “depression physical activity”), and treatments for depression in patients with CVD (eg, “depression medication CVD,” “cognitive behavioural therapy depression cardiac surgery”). We reviewed titles and abstracts from these searches, obtained full-text articles for relevant studies, and examined reference lists to identify additional relevant manuscripts.

## DEPRESSION

Depression is a mood disorder marked by a persistent sense of sadness and/or an inability to experience pleasure, often accompanied by impairments in daily functioning. In its most severe form, depression is associated with suicide.^7^ Globally, depression is a major cause of disability and a significant contributor to the loss of productive years of life.^2^ According to the American Psychiatric Association (2013), several well-established risk factors contribute to the likelihood of developing depression. These include low socioeconomic status (SES) and the presence of comorbid chronic medical conditions such as CVD, diabetes, or obesity. Additionally, a family history of depression or a personal history of the condition significantly increases the risk of experiencing depression.

## DIAGNOSIS OF DEPRESSION

MDD is formally diagnosed using the Diagnostic and Statistical Manual of Mental Disorders fifth edition criteria (DSM-5) (**Table 1**) and through a clinical interview, with symptom severity usually measured using clinical ratings or psychometric questionnaires. Commonly used psychometric questionnaires are: Beck Depression Inventory-II (BDI-II), Centre for Epidemiological Studies-Depression Scale (CES-D), Hospital Anxiety, Depression Scale-Depression (HADS-D), and The Patient Health Questionnaire (PHQ).^8^

**Table 1.** Diagnostic Criteria for Major Depressive Disorder according to DSM-5

Five or more of the following A criteria (at least 1 includes A1 or A2)
A1 Depressed mood—indicated by subjective report or observation by others.
A2 Loss of interest or pleasure in almost all activities—indicated by subjective report or observation by others.
A3 Significant (>5 % in a month) unintentional weight loss/gain or decrease/increase in appetite.
A4 Sleep disturbance (insomnia or hypersomnia).
A5 Psychomotor changes (agitation or retardation) severe enough to be observable by others.
A6 Tiredness, fatigue, or low energy, or decreased efficiency with which routine tasks are completed.
A7 A sense of worthlessness or excessive, inappropriate, or delusional guilt (not merely self-reproach or guilt about being sick).
A8 Impaired ability to think, concentrate, or make decisions—indicated by subjective report or observation by others.
A9 Recurrent thoughts of death (not just fear of dying), suicidal ideation, or suicide attempts.
The symptoms cause clinically significant distress or impairment in social, occupational, or other important areas of functioning.
The symptoms are not due to the direct physiological effects of a substance (eg, drug abuse) or a medical condition (eg, hypothyroidism).
There has never been a manic episode or hypomanic episode.
Major depressive episode is not better explained by schizophrenia spectrum or other psychotic disorders.

Symptom must persist most of the day, daily, for at least 2 weeks in a row, excluding A3 and A9.

## SCREENING

Patient-reported outcome measures are valuable in both pre- and post-surgical settings, as they help identify at-risk patients, guide referrals to psychological interventions, and assess treatment efficacy.^9^^,^^10^ Routine screening for depression and anxiety during assessments and follow-ups has been widely advocated to monitor psychological distress and support patient-centred care.^9^^,^^10^ Furthermore, integrating clinical psychologists into cardiovascular care teams is important for addressing psychological factors within a multidisciplinary framework.^11^^,^^12^ The American Heart Association (AHA) has published a scientific statement highlighting the crucial role of psychological well-being in cardiovascular health and stresses the importance of screening for depression in individuals with CVD.^13^

Even so, routine screening for depression before cardiac surgery is uncommon, despite its clear impact on outcomes. Key barriers include limited time in preoperative clinics, lack of clinician training in using or interpreting screening tools, unclear referral pathways, and minimal integration of mental health services within cardiac units.^11^ Practical solutions include embedding screening tools, such as the PHQ-9, into standard pre-op assessments to reduce burden.^13^ Training staff on screening relevance and establishing clear follow-up protocols can facilitate uptake. Developing partnerships with psychology or psychiatry services ensures timely evaluation and care.^11^

## LIFESTYLE AND BEHAVIOUR

Negative health behaviours, such as poor diet, obesity, physical inactivity, substance abuse, and smoking, are well-known contributors to chronic medical conditions like CHD, type 2 diabetes, and hypertension.^14^ Psychological factors are also linked to these negative health behaviours.^15^ Research indicates that individuals with depression are more likely to smoke, be physically inactive, overweight, and have higher rates of hypertension, diabetes, and metabolic syndrome compared to non-depressed individuals.^16^ Additionally, smokers with depression tend to smoke more cigarettes daily and are less likely to quit smoking than those without depression.^17^ The bidirectional relationship between physical health behaviours and psychological well-being suggests that harmful health behaviours can negatively affect psychological well-being.^15^ Conversely, while anxiety and anger are normal conditions/feelings and can be functional for humans in appropriate doses, persistent high levels of these conditions/feelings can be dysfunctional. This dysfunction can negatively impact adherence to health-promoting behaviours and, in turn, increase the risk of CVD.^17^

## SOCIOECONOMIC STATUS

Low SES has been associated with depression and is recognized as a risk factor for CVD and higher rates of all-cause mortality.^18^ It is worth remembering that the relationship between SES and CVD and cardiovascular risk factors has changed over time. In the early 20th century, individuals with higher SES experienced a greater burden of CVD, probably due to a lifestyle that allowed smoking, unhealthy diets, and sedentary lifestyles to a larger extent than in individuals with lower SES. However, as demographic, epidemiological, and nutritional transitions progressed, and significant improvements were made in CVD prevention and treatment, the burden of CVD shifted towards individuals with lower SES.^19^ The association between lower SES and increased mortality is particularly evident in countries lacking universal healthcare coverage. However, in Sweden, a country with universal tax-funded healthcare, a large study conducted by Dalén et al^20^ demonstrated that lower SES, as measured by household disposable income, was still associated with increased all-cause mortality following cardiac surgery. There was a stepwise inverse association between household disposable income and all-cause mortality. They also noted that patients with a lower household disposable income had a higher prevalence of cardiovascular risk factors and differed in educational level, civil status, and birth region compared to those in higher income groups.^20^ Socioeconomic factors have been associated with depression. Murphy et al^21^ demonstrated that financial strain significantly increases the risk of both early and late depression by 4-5 times. Similarly, Dunkel et al^22^ reported that a low educational level is linked to a nearly 2-fold increased risk of depression in patients undergoing coronary artery bypass grafting (CABG), with an odds ratio (OR) of 1.72 (95% CI, 1.17-2.53).

## SOCIAL DEPRIVATION/LONELINESS

Social deprivation refers to the inability of individuals to participate in their personal or societal life fully. The measurement of social deprivation often emphasizes the lack of material or financial resources, subsequently contributing to reduced social participation.^23^ Risk factors such as smoking, obesity, and diabetes are associated with social deprivation and are linked to poorer prognosis after cardiac surgery.^23^ Loneliness, a consequence of social deprivation, negatively affects patients with cardiac diagnoses and are also linked to the risk of CVD.^16^ A Danish study found that feelings of loneliness were associated with poor patient-reported outcomes and higher 1-year mortality rates in both men and women across cardiac diagnoses.^24^ For individuals undergoing CABG, low family support, or living alone is associated with symptoms of depression, anxiety, and hopelessness during the postoperative follow-up period.^24^ Chongopklang et al^25^ further identified that limited social support, diminished hope, negative illness perceptions, and preoperative anxiety are predictors of depression following CABG.

## DEPRESSION AND SECONDARY PREVENTION OF CARDIOVASCULAR DISEASE

Depression is associated with increased short- and long-term mortality, stroke, and rehospitalization, following cardiac surgery.^6^ Secondary prevention of CVD after cardiac surgery is of utmost importance, and depression could have a negative impact on secondary prevention. Behavioural phenomena commonly observed in depressed patients, such as social isolation, feelings of hopelessness, and a lack of belief in the value of activities, could serve as barriers to secondary preventive efforts; for example, persons with depression may fail to adhere to prescribed medicines or may be unable to collect prescriptions. Persons with depression have lower rates of medication adherence and are less prone to make lifestyle changes after myocardial infarction.^26^ Adherence to secondary prevention medications after cardiac surgery in persons with depression is sparsely studied, but 1 large study found that depressed persons were prescribed secondary prevention medications at the same rate as non-depressed people and also filled prescriptions at the same rate.^27^

## PREVALENCE AND CONSEQUENCES OF PREOPERATIVE DEPRESSION IN PATIENTS WITH CVD UNDERGOING CARDIAC SURGERY

The prevalence of depression varies through life, and according to data from the National Health and Nutrition Examination Survey, depression rates in the United States are 10.1% among women and 5.5% among men aged 20 to 39 years, 11.5% versus 5.2% for those aged 40 to 49 years, and 9.6% versus 6.1% for individuals aged 60 years and above. These statistics highlight the varying impact of depression across different age groups and genders.^2^

**Table 2** demonstrates the prevalence of depression among cardiac surgery populations and the diagnostic methods used to measure depressive symptoms. Several studies show, like in the general population, a higher prevalence of depression among women compared to men undergoing cardiac surgery.^5^^,^^28^^,^^29^

**Table 2.** Summary of Selected Studies Investigating Depression and Survival in Cardiac Surgery

First author (year)	Country	Study period	Study population	Total number of patients (depression/control)	Definition of depression	Outcome^a^	Follow-up time, years	Major finding
Blumenthal (2003)	United States	1989-2001	CABG	817 (97/720)	CES-D > 27 (moderate to severely depressed)	All-cause mortality	Median: 5.0	Depression associated with mortality (HR: 2.4)
Ho (2005)	United States	1992-1996	Valve surgery ± CABG	648 (189/489)	MHI-5 depression screen	All-cause mortality	0.5	Depression associated with 6 months mortality (OR: 1.9)
Tully (2008)	Australia	1996-2006	CABG	440 (89/351)	Self-report DASS ≥10	All-cause mortality	Median: 5.9	Depression not associated with mortality
Tully (2012)	Australia	1996-2008	CABG	4136 (105/4031)	SSRI/SNRI use from hospital database	In-hospital complications and long-term all-cause mortality	Median: 4.7	Depression associated with renal dysfunction and prolonged ventilation, but not bleeding events or long-term mortality
Xiong (2006)	United States	1999-2003	CABG	4794 (246/4548)	SSRI use from inpatient pharmacy records	All-cause mortality and rehospitalization	Median: 3.0	Depression associated with all-cause mortality (HR: 1.6)
Stenman (2013)	Sweden	2006-2008	CABG	10884 (1171/9713)	Antidepressant use from national prescribed drug register	All-cause mortality and rehospitalization	Mean: 3.5	Depression associated with all-cause mortality (HR: 1.4)
Stenman (2014)	Sweden	1997-2008	CABG	56064 (324/55740)	ICD-10 codes from national hospital discharge register	All-cause mortality and rehospitalization	Mean: 7.5	Depression associated with all-cause mortality (HR: 1.6), and a combination of death or rehospitalization for heart failure, myocardial infarction, or stroke (HR: 1.6)
Falk (2024)	Sweden	2013-2016	Valve surgery, CABG, other	1120 (162/958)	PHQ-9 ≥ 10	All-cause mortality	Mean: 7.2	Self-reported depressive symptoms associated with all-cause mortality (HR: 1.7)

a All studies used statistical approaches to account for confounding.

Abbreviations: CABG: coronary artery bypass graft; CES-D: 20-item Centre for Epidemiological Studies Depression questionnaire; DASS: Depression Anxiety Stress Scales; HR: hazard ratio; MHI: mental health inventory; OR: odds ratio; PHQ: Patient Health Questionnaire; SNRI: Serotonin-norepinephrine reuptake inhibitor; SSRI: Selective serotonin reuptake inhibitor.

## PREOPERATIVE DEPRESSION AND POSTOPERATIVE NEUROCOGNITIVE DISORDERS

Postoperative neurocognitive disorders, including postoperative cognitive dysfunction (POCD) and postoperative delirium (POD), are common after cardiac surgery. A recent study on CABG patients found that 25% developed POD and 32.5% developed POCD.^30^

POCD is a temporary or prolonged impairment of cognitive functions following surgery, including perception, information processing, and memory. While typically transient, POCD can persist from weeks to several months postoperatively. Preoperative depression has, along with other risk factors such as advanced age and cardiopulmonary bypass, been associated with POCD after cardiac surgery.^31^ POD is prevalent in patients undergoing cardiac surgery and can lead to various complications, including longer hospital stays, poorer long-term outcomes, and reduced quality of life for patients.^32^ According to the DSM-5 criteria symptoms of POD include rapid onset and fluctuating attention disturbances, confusion, and sleep disturbances. In a population-based cohort study including 1120 patients, Falk et al found that 14% of patients reported depressive symptoms before surgery. The incidence of POD was 26%, with the highest rates observed among elderly patients. Among those with preoperative depression, 34% developed POD, compared to 24% in the non-depressed group. The adjusted odds of developing delirium were 2.19 times higher (95% confidence interval: 1.43-3.34) in individuals with depressive symptoms than in those without.^33^

## PREOPERATIVE DEPRESSION AND MORTALITY

Depression is common in individuals with CVD and in patients undergoing cardiac surgery. Clinically significant depression is present in 31%-45% of patients with coronary artery disease.^34^ It has been hypothesized that inflammation is a common link between CVD and depression,^35^ which is why most studies have focused on patients undergoing CABG. In one of the first studies on this subject, Blumenthal et al used the Centre for Epidemiological Studies-Depression (CES-D) scale to assess preoperative depression in patients undergoing CABG. They found a prevalence of depression of 38% and determined that depression was an important independent predictor of death after CABG, with an adjusted hazard ratio (HR) of 2.4 (95% CI, 1.4-4.0).^5^ In a study by Xiong et al, preoperative depression was assessed using selective serotonin reuptake inhibitors (SSRIs) as a proxy for depression. They found that only 5.1% of the patients used SSRIs before CABG, but there was still an association with a higher risk of postoperative mortality and rehospitalizations (HR 1.61; 95% CI, 1.17-2.21).^36^

Later, Tully et al continued this line of research and used the Depression Anxiety Stress Scale (DASS) to identify preoperative depression and anxiety among patients undergoing CABG. They found no association between depression and an increased risk of mortality after cardiac surgery, however, there was an increased risk of mortality among those with preoperative anxiety.^37^ In a subsequent study, Tully et al used SSRI/serotonin norepinephrine reuptake inhibitor (SNRI) use as a proxy for depression, but no association could be confirmed between the use of antidepressants and mortality after CABG. However, there was a significant association between depression and renal dysfunction and prolonged ventilation after CABG.^38^ Stenman et al explored the relationship between depression and mortality after CABG in 2 large national cohort studies. In the first study, antidepressant use served as a proxy for depression, revealing a significant association with long-term mortality after CABG (HR 1.45; 95% CI, 1.18-1.77).^39^ Although the use of antidepressant medication as a proxy for depression is a pragmatic approach in several studies, it has inherent limitations. Further research is needed to clarify the underlying mechanisms and establish causal relationships between depression and cardiovascular outcomes. In a second study, Stenman et al^6^ used ICD codes to identify depression, indicating an even stronger association with long-term mortality (HR 1.65; 95% CI, 1.37-1.99). Similar results were identified in a study including patients undergoing valve surgery.^40^

In a Swedish prospective cohort study, preoperative depression was evaluated with the PHQ-9 in patients undergoing various kinds of cardiac surgery. This is one of the first studies to investigate the long-term association between preoperative depression and mortality in patients undergoing surgical cardiac procedures not limited to ischaemic heart disease, and the results showed a significant association (HR 1.66; 95% CI, 1.09-2.54).^29^

## POSTOPERATIVE DEPRESSION

In a Swedish national register-based study involving 125 418 isolated CABG patients and 495 371 age- and sex-matched individuals from the general population, the cumulative incidence of new-onset depression was 6.1% and 4.7%, respectively. New-onset depression after CABG was most prominent in men below 65 years during the first 5 years after surgery.^41^ Horne et al^42^ also studied pre- and postoperative depression in cardiac surgery patients, using the PHQ-9 questionnaire to identify preoperative and postoperative depression in patients undergoing cardiac surgery. They found that the prevalence of depression was 23% preoperatively and increased to 38% postoperatively and that depressive symptoms prolonged hospital stays by more than 7 days. Preoperative depression was also associated with the highest risk for postoperative depression.^42^ These findings highlight that depression is common in patients undergoing cardiac surgery, both before and after surgery, and should be evaluated.

## SEX AND GENDER DIFFERENCES

The causes of depression are not fully understood but are likely a complex mix of genetic, biological, environmental, inflammatory, and psycho-social factors.^43^ Depression is observed to be twice as common in women compared to men.^43^ These variations in depression rates may be influenced by both biological sex and gender, the latter being a socially constructed concept not rooted in biology. There are studies indicating significant sex differences in depression-related gene expression, neuroplasticity, and immune signatures, which may contribute to the disparities in the prevalence and pathoetiology of depression between men and women.^43^ However, gender also plays a role. Typical symptoms of depression, such as feelings of hopelessness, loss of interest or pleasure in activities, fatigue, and lack of energy, are more commonly reported by women. In contrast, men often exhibit symptoms like irritability, overworking, substance misuse, and aggression.^44^ Although men are diagnosed with depression at about half the rate of women, they die by suicide 3-4 times more often.^45^ Since depression is a significant risk factor for suicide, it is possible that many men with depression go undiagnosed. When a study included these “male-typical” symptoms of depression, the difference in depression rates between men and women disappeared, suggesting that depression in many men often goes unrecognized.^44^

## PATHOPHYSIOLOGY

The precise mechanism by which depressive symptoms may increase cardiovascular risk is not fully understood. Various pathophysiological pathways have been recognized; for example, patients with depression often exhibit high sympathetic tone, hypercortisolemia, elevated catecholamine levels, increased inflammatory markers, and abnormal platelet activation.^16^ Additionally, factors such as lifestyle behaviours and gender play a role in the development of depression. The relationship between depression and CVD is therefore complex and multifactorial.^43^

### Inflammatory markers

Chronic inflammation is a key factor in the development of several chronic disorders, including CVD. Harmful stimuli like cigarette smoke, LDL cholesterol, and hypertension damage the arterial wall, leading to arterial inflammation.^46^ Additionally, small proteins known as cytokines influence cell functions and interactions. They are part of the immune system and can have either pro-inflammatory or anti-inflammatory effects.^47^^,^^48^ In several meta-analyses, it has been established that proinflammatory cytokines and acute phase proteins are elevated in patients with depression, and levels of IL-6, TNF, and C-reactive protein (CRP) have been shown to be higher in the blood of depressed patients compared to healthy controls.^47^^,^^49^^,^^50^ This inflammatory state may significantly contribute to the increased risk of coronary artery disease in individuals with depression.

### Endothelial dysfunction

Endothelial dysfunction has been hypothesized to mediate the increased risk of coronary artery disease in patients with depression. In addition to delivering nutrients and oxygen, the endothelium plays a crucial role in immune regulation and maintaining homeostasis.^51^ Endothelial cells are connected to adjacent cells through paracellular junctional adhesion molecules, forming a tight barrier that prevents blood contents from leaking into tissue parenchyma.^52^ The apical membrane of endothelial cells is also lined with adhesion molecules that bind circulating immune cells and platelets. The regulation of these adhesion molecules significantly impacts immune cell trafficking, inflammation, and the progression of vascular diseases.^52^ Studies have shown that mental stress is linked to endothelial function. One study showed that acute psychological stress caused temporary endothelial dysfunction in healthy men without known cardiovascular risk factors,^53^ and in another study involving healthy individuals without cardiovascular risk factors, a 3-minute mental stress task led to prolonged endothelial dysfunction.^54^

### Platelet activation

Numerous clinical studies have demonstrated an increase in platelet activation markers in patients with depression.^55^ For instance, a study involving 300 patients with both CVD and depression found that these patients had higher serotonin receptor levels and more platelet aggregation compared to those with only depression.^56^ Similarly, other studies have confirmed that patients with coronary artery disease and depression exhibit elevated platelet specific volume and higher levels of platelet activation markers.^57^ It is suggested that depression-induced platelet activation releases factors that exacerbate atherosclerosis progression, thereby increasing the risk of adverse cardiovascular events.^58^

## TREATMENT

The 2024 EACTS guidelines on perioperative medication in adult cardiac surgery recommend that continuation of antidepressants, particularly SSRIs and SNRIs, should be considered during the perioperative period in patients already on treatment, and support depression screening and careful monitoring of psychiatric medications to optimize both mental health and surgical outcomes.^59^

### Cardiac psychiatry/behavioural cardiology

Biopsychosocial evaluations analyse biological, psychological, and social factors in combination to understand a patient and to guide treatment decisions. Regardless of the remaining uncertainties concerning the possibility that CVD and mental illness share underlying pathophysiologic mechanisms, individualized treatment regimens based on multidisciplinary assessments are likely favourable. Pharmacological and psychological treatment of mental illness aims to jointly alter human behaviour in order to increase mental and physical functioning and alleviate symptoms.^60^

### Cardiovascular and psychiatric medication

The aforementioned bidirectional relationship between mental illness and CVD also applies from a pharmacological standpoint. In patients with CVD, the use of aspirin and statins has been associated with a lower risk of depression, whereas calcium channel blockers, diuretics, nitrates, and β-blockers have been associated with a higher risk of depression.^61^ Depression has been shown to increase the risk of cardiac arrythmias and sudden cardiac death.^62^ In addition, some psychotropic drugs have proarrhythmic properties, including antidepressants and second-generation antipsychotics, which can be used as augmenting agents in the treatment of MDD.^63^

### Antidepressants and arrythmia

The arrhythmogenic effect of tricyclic antidepressants (TCAs) has been well established and associated with the prolongation of PR, QRS, and QT interval.^64^ The use of TCAs is commonly not recommended in CVD patients at risk of arrythmia but can be used in special care settings with proper monitoring. By contrast, the arrhythmogenic effect of non- TCAs (ie, SSRIs, SNRIs, noradrenergic and specific serotonergic antidepressants [NaSSAs], noradrenergic and dopamine reuptake inhibitor [NDRIs], and others, see **Table 3**) is generally considered to be low. Overall, SSRIs have been associated with modest QT interval changes. However, the effect seems to be negligible at recommended clinical doses.^65^ Vortioxetine and agomelatine have not been shown to cause QT prolongation.^66^ Sertraline and mirtazapine are considered safe in patients with ischaemic heart disease.^67^ The use of citalopram, escitalopram, venlafaxine, and bupropion warrants closer monitoring in CVD patients at risk of serious arrythmias.^68^

**Table 3.** Cardiac Effect of Antidepressants—Modified from The Maudsley Prescribing Guidelines in Psychiatry, 14th Edition, David M Taylor, Thomas R. E. Barnes, Allan H. Young, Wiley Blackwell (2021), ISBN: 978-1-119-77222-4

Drug	Heart rate	Blood pressure	QTc	Arrhythmia	Conduction disturbance	Licensed restrictions post MI	Comments
Agomelatine	No changes reported	No changes reported	Single case of QTc prolongation	No arrhythmia reported	Unclear	No specific contra-indication	Cautiously recommended
Bupropion	Slight increase	Slight increase in blood pressure but can sometimes be significant. Rarely postural hypotension	QTc shortening, but prolongation has been reported in cases of overdose	No effect. Reports in overdose	None	Well tolerated for smoking cessation in post MI patients	Be aware of interaction potential. Monitor blood pressure
Citalopram (assume same for escitalopram)	Small decrease in heart rate	Slight drop in systolic blood pressure	Dose-related increase in QTc	Torsade de Pointes reported, mainly in overdose	None	Caution in patients with recent MI or uncompensated heart failure. But some evidence of safety in CVD	Minor metabolic which increases QTc interval. No clear evidence of increased risk of arrhythmia at any licensed dose
Duloxetine	Slight increase	Important effect. Caution in hypertension	Isolated reports of QT prolongation	Isolated reports of toxicity	Isolated reports of toxicity	Caution in patients whose conditions could be compromised by an increased heart rate or by an increase in blood pressure	Not recommended in cardiac disease
Fluoxetine	Small decrease in heart rate, minimal effect on blood pressure		No effect on QTc interval	None	None	Caution in patients with acute MI or uncompensated heart failure	Evidence of safety post MI
Fluvoxamine	Minimal effect on heart rate	Small drop in systolic blood pressure	No significant effect on QTc	None	None	Caution	Limited changes in ECG have been observed
Levomilnacipran	Slight increase	Small increase	No effect on QTc interval	Pre-existing tachyarrhythmias should be treated before initiating treatment	None	Caution in cardiac patients	Monitor heart rate and blood pressure
Lofepramine	Modest increase in heart rate	Less decrease in postural blood pressure compared with other TCAs	Can possibly prolong QTc interval at higher doses	May occur at higher doses, but rare	Unclear	Caution in patients with recent MI	Less cardiotoxic than other TCAs. Reasons unclear
MAOIs	Decrease in heart rate	Postural hypotension. Risk of hypertensive crisis	Unclear but may shorten QTc interval at higher doses	May cause arrhythmia and decrease LVEF	No clear effect on cardiac conduction	Use with caution in patents with cardiovascular disease	Not recommended in CV patients
Milnacipran	Slight increase in heart rate	Small increases in systolic and diastolic BP	No effect on QTc	None	None	Caution	Avoid in hypertension and heart failure
Mirtazapine	Minimal change in heart rate	Minimal effect on blood pressure	No effect on QTc	None	None	Caution patients with recent MI	Evidence of safety post MI. Good alternative to SSRIs
Moclobemide	Marginal decrease in heart rate	Minimal effect on blood pressure. Isolated cases of hypertensive episodes	No effect on QTc interval in normal doses. Prolongation in overdose	None	None	General caution in patients with a history of cardiac disorders	Possibly arrhythmogenic in overdose
Paroxetine	Small decrease in mean heart rate	Minimal effect on blood pressure	No effect on QTc interval	None	None	General caution in cardiac patients	Probably safe post MI
Reboxetine	Significant increase in heart rate	Marginal increase in systolic and diastolic blood pressure. Postural decrease at higher doses	No effect on QTc	Rythm abnormalities may occur	Atrial and ventricular ectopic beats, especially in the elderly	Caution in patients with cardiac disease	Probably best avoided in coronary disease
Sertraline	Minimal effect on heart rate	Minimal effect on blood pressure	No effect on QTc interval at standard doses. Small increase (>10 ms) at 400 mg/day	None	None	Drug of choice post MI but formal labelling acknowledges effect on QT cautions against use in patients with additional risk factors for QTc prolongation	Safe post MI an in heart failure
Tricyclics	Increase in heart rate	Postural hypotension	Prolongation of QTc interval and QRS interval	Ventricular arrhythmia common in overdose. Torsade de Pointes reported	Slows cardiac conduction—blocks cardiac Na/K channels	Contraindicated in patients with recent MI	TCAs affect cardiac contractility. Some TCAs linked to ischaemic heart disease and sudden cardiac death. Avoid in coronary artery disease
Venlafaxine (assume same for desvenlafaxine)	Marginally increased	Some increase in postural blood pressure. At higher doses increase in blood pressure	Possible prolongation in overdose, but very rare	Rare reports of cardiac arrhythmia in overdose	Rare reports of conduction abnormalities	Has not been evaluated in post MI-patients. Caution advised	Evidence for arrhythmogenic potential is slim, but avoid in coronary disease
Vortioxetine	No effect	No effect	No effect	No effect	No effect	No specific contraindication	Trial data suggest no effect on QTc or coagulation parameters

Abbreviations: CVD: cardiovascular disease; ECG: electrocardiography; MAOI: monoamine oxidase inhibitors; MI: myocardial infarction; SSRI: selective serotonin reuptake inhibitor; TCA: tricyclic antidepressants.

### Drug-drug interactions

Pharmacokinetic drug interactions resulting in sub-therapeutic or toxic effects are in this context often mediated by inhibition or induction of CYP450 enzymes. The efficacy of some of these enzymes is also subject to genetic and environmental influence (eg, tobacco smoke, grapefruit juice) and sometimes difficult to predict.^69^ Pharmacodynamic interactions should also be considered. For example, TCAs can cause postural hypotension via adrenergic ⍺1-recpetor blockage, SSRIs inhibit platelet aggregation and can induce hyponatremia, and SNRIs may increase blood pressure.^70^ Taken together, untreated depression is a risk factor for poorer prognosis in patients with CVD.^71^

## MULTIDISCIPLINARY APPROACH

**Figure 1.**
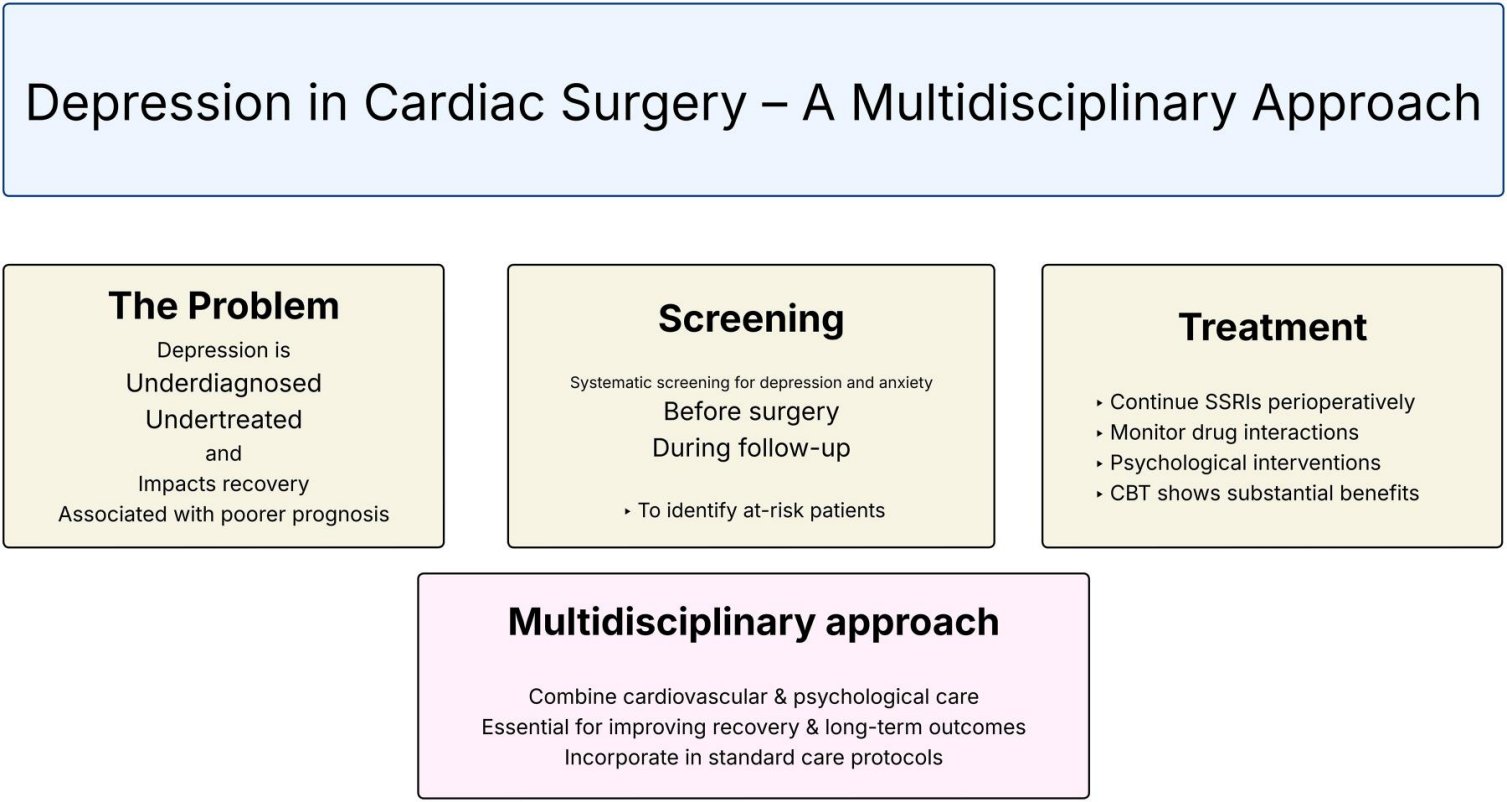
Depression in Cardiac Surgery—A Multidisciplinary Approach

Early detection of concomitant mental illness in patients with CVD and a multidisciplinary biopsychosocial approach to guide treatment decisions is likely favourable. A judicious use of antidepressants, when indicated, in conjunction with close monitoring of potential adverse events, has the potential to improve quality of life and prognosis (**Figure 1**).

## PSYCHOLOGICAL THERAPIES FOR DEPRESSION AND CARDIOVASCULAR RISK

Meta-analyses demonstrate that cognitive behavioural therapy (CBT) is an effective intervention for improving psychological outcomes, such as depression and anxiety, in patients with CHD and other cardiovascular conditions^72^^,^^73^ Additionally, CBT has been shown to have a positive impact on physical health outcomes, including enhanced cardiac function and reduced hospitalization rates, emphasizing its potential as an adjunctive intervention in cardiac rehabilitation.^72^^,^^73^ Evidence further supports CBT as a first-line treatment for anxiety and depression, with comparable efficacy observed between online and traditional delivery formats.^74^ Due to the scalability and accessibility of online CBT, the approach offers a promising solution for widespread clinical implementation and has been shown to be beneficial in reducing psychological distress and healthcare consumption in cardiac populations.^75^ A 2015 systematic review and meta-analysis demonstrated that psychological interventions significantly reduce depression and anxiety in cardiac surgery patients (CABG, ICD, other; *N* = 2718).^76^ Interventions initiated pre- or post-surgery and tailored to individual needs yielded the greatest benefits, emphasizing the value of personalized approaches to optimal mental health outcomes.^76^ Several randomized controlled trials (RCTs) have demonstrated the potential of CBT to alleviate depression and support enhanced recovery in this population. For example, 1 RCT demonstrated that CBT combining cognitive restructuring, behavioural activation, and psychoeducation significantly reduced depressive symptoms compared to usual care.^77^ Similarly, another RCT highlighted that a structured CBT program incorporating stress management and problem-solving strategies led to substantial reductions in depressive symptoms after CABG surgery, emphasizing its potential role in post-surgical recovery.^78^ Additionally, CBT has been shown to improve pain perception and management, with interventions such as relaxation techniques and cognitive restructuring enhancing both pain control and patient outcomes compared to standard care. Furthermore, CBT has been linked to improved physiological outcomes, including enhanced heart rate variability, indicating a potential connection between psychological interventions and autonomic function. A recent pilot study, which included a 5-week internet-delivered CBT intervention targeting cardiac anxiety and depressive inactivity following cardiac surgery, demonstrated feasibility, high adherence, and significant improvements in depressive symptoms, cardiac anxiety, and perceived severity of postoperative symptoms.^79^ These findings underscore the potential for scalable, brief psychological interventions to enhance recovery and reduce postoperative distress in cardiac surgery patients, warranting further evaluation in an RCT.

These studies highlight the potential of CBT in addressing psychological distress, depression, and behavioural factors that may hinder recovery, ultimately enhancing rehabilitation and improving overall patient outcomes.

In a clinical setting, it would be desirable for clinical psychologists to be integral members of cardiac surgery care teams, providing routine clinical services, treatment, follow-up, and consultation to medical staff.^11^ Multidisciplinary approaches that include psychologists have shown benefits for quality of life, self-management, cardiac outcomes, and healthcare use in cardiac patients.^11^ Establishing clear referral protocols to in-house or affiliated mental health providers could help ensure that patients with elevated symptoms of depression receive appropriate care. Incorporating these processes into preoperative and postoperative checklists and clinical workflows could further support the care team and facilitate integration into routine surgical care.

## EFFECT OF EXERCISE ON DEPRESSION

Exercise is a sub-category of physical activity and should be performed in a structured way to maintain or improve aerobic capacity or muscular strength. Exercise as treatment in patients with MDD has proven to be as beneficial as medication with SSRI and to even lower rates of depression after 6-month follow-up.^15^ In a recent systematic review and network meta-analysis of RCTs, Noetel et al^80^ confirmed that exercise is an effective treatment for depression. Walking or jogging, yoga, and strength training are particularly effective forms of exercise for treating depression, especially when performed intensely. They also found exercise to be equally effective for individuals with and without comorbidities and across different baseline levels of depression.^80^

For persons with depression who have undergone cardiac surgery, getting started with exercise can be challenging. This is partly because the depression itself can make it difficult to get started or get out among other people, but also because of the anxiety that arises after cardiac surgery (eg, how much does the patient dare to take on, concerns about whether exercise can actually damage the heart, etc.). Therefore, it is particularly important that the postoperative information to patients is clear about the recommendations that apply, and that time is given to answer questions about exercise after surgery.

## CONCLUSION

Depression is associated with poorer prognosis after cardiac surgery and is associated with higher rates of re-hospitalizations, prolonged hospital stays, impaired postoperative cognitive function and quality of life—factors that can lead to further complications and compromise surgical outcomes.

## TAKE-HOME MESSAGES

- Depression is often underdiagnosed and undertreated among cardiac surgery patients.
- Depression significantly impacts recovery in patients undergoing cardiac surgery and is linked to a higher risk of worse outcomes.
- Continuation of SSRIs during the perioperative period should be considered for patients already receiving treatment, with close attention to potential drug interactions.
- Systematic psychological screening for depression and anxiety should be implemented both before surgery and during follow-up to identify at-risk patients.
- Psychological interventions, particularly CBT, have been shown to have substantial benefits.
- A multidisciplinary approach that combines cardiovascular and psychological care is essential for improving recovery and long-term outcomes.
- By incorporating psychological screening and interventions into standard care, healthcare systems can enhance postoperative outcomes, reduce complications, and provide more comprehensive support for cardiac surgery patients

## GAPS IN KNOWLEDGE

It is essential to clarify the underlying mechanisms linking depression and CVD. Longitudinal studies are needed to establish a causative rather than only correlational relationship.

## FUTURE RESEARCH DIRECTIONS

Future research directions include evaluating the treatment of depression, examining gender differences, and determining whether men and women should receive the same type of treatment. Additionally, it is important to investigate the optimal timing and type of treatment to be administered in conjunction with surgery for patients with depression. Further research is necessary to evaluate the effectiveness of psychological interventions for depression in cardiac surgery patients. More clinical trials are needed, particularly within real-world care systems, to assess their generalizability across diverse populations and healthcare settings.

## Data Availability

No new data were generated or analysed in support of this research.
